# Biology of Healthy Aging: Biological Hallmarks of Stress Resistance Related and Unrelated to Longevity in Humans

**DOI:** 10.3390/ijms251910493

**Published:** 2024-09-29

**Authors:** Komalpreet Badial, Patricia Lacayo, Shin Murakami

**Affiliations:** Department of Foundational Biomedical Sciences, College of Osteopathic Medicine, Touro University California, Vallejo, CA 94592, USA

**Keywords:** aging, longevity, stress damage, stress resistance, stress resilience, metabolism, progeria, insulin, endocrine, nuclear integrity

## Abstract

Stress resistance is highly associated with longer and healthier lifespans in various model organisms, including nematodes, fruit flies, and mice. However, we lack a complete understanding of stress resistance in humans; therefore, we investigated how stress resistance and longevity are interlinked in humans. Using more than 180 databases, we identified 541 human genes associated with stress resistance. The curated gene set is highly enriched with genes involved in the cellular response to stress. The Reactome analysis identified 398 biological pathways, narrowed down to 172 pathways using a medium threshold (*p*-value < 1 × 10^−4^). We further summarized these pathways into 14 pathway categories, e.g., cellular response to stimuli/stress, DNA repair, gene expression, and immune system. There were overlapping categories between stress resistance and longevity, including gene expression, signal transduction, immune system, and cellular responses to stimuli/stress. The categories include the PIP3-AKT-FOXO and mTOR pathways, known to specify lifespans in the model systems. They also include the accelerated aging syndrome genes (WRN and HGPS/LMNA), while the genes were also involved in non-overlapped categories. Notably, nuclear pore proteins are enriched among the stress-resistance pathways and overlap with diverse metabolic pathways. This study fills the knowledge gap in humans, suggesting that stress resistance is closely linked to longevity pathways but not entirely identical. While most longevity categories intersect with stress-resistance categories, some do not, particularly those related to cell proliferation and beta-cell development. We also note inconsistencies in pathway terminologies with aging hallmarks reported previously, and propose them to be more unified and integral.

## 1. Introduction

Aging is a complex biological process characterized by gradual changes, including mostly declines in biological function and increased vulnerability to external and internal stress over time. The age-related changes and modifications occur in macromolecules, leading to genetic and epigenetic changes at multidimensional levels, ranging from molecules, cells, extracellular regions, tissues, and the whole body. The major challenge in the field of aging has been to identify the causes of aging and to distinguish their consequences. Herman (1956) focused on oxidative stress, such as reactive oxygen species, namely ROS (OH and HO_2_), as a possible cause of aging (the free radical theory of aging) [1]. Mutations in ROS scavenging enzymes, such as superoxide dismutase genes, however, have little or modest effects on lifespans [2,3,4,5]. With a wide variety of ROS functions, it has been experimentally difficult to prove that oxidative stress is the sole cause of aging despite its deleterious nature as a single stress, and mounting evidence suggests that oxidative stress may play more specific roles in age-related diseases [6,7]. Similarly, thermotolerance dependent on HSF-1 can be separated from life extension in worms [8]. Importantly, genetic and pharmacological screenings for single-stress resistance (resistance to a single form of stress) have yielded those that confer life extension and those that do not confer life extension [9,10,11], suggesting two types of stress resistance, longevity-related and longevity-unrelated. Thus, considering single stress as a sole cause of aging has limitations.

In the model systems, including worms and mice, life-extending mutants show increased resistance to various stressors, including oxidative stress, heat, ultraviolet light, and DNA-damaging agents, among others [Reviewed in Refs. [12,13,14,15]]. This resistance to multiple forms of stress (multiplex stress response and resistance, or multiplex stress resistance, in short) is more associated with life extension. The original concept of multiplex stress resistance was first introduced, reading: “The finding that all Age (life-extending) mutations show resistance to multiple distinct stresses suggests the possibility that a common molecular mechanism may regulate the response to all three stressors (oxidative stress, heat and UV) [16]”. The term multiplex stress resistance was later defined when the finding was extended to the cells of long-lived dwarf mice [17,18]. Furthermore, multiplex stress resistance is reduced during aging in worms [5], and the pathways for stress responses are needed for life extension [13,15]. However, stress resistance does not explain all aspects of aging [19,20], which suggests the transitional phase before advanced aging [20,21,22]. Taken together, we still do not have a clear overview of what biological pathways contribute to stress resistance in humans and how they are related to longevity.

In addition to life-extending interventions, the recent discussions about halting or reversing aging have sparked hope for mitigating the negative effects of aging. These discussions may be divided into two main groups. Firstly, certain wild species, such as turtles, eight bird species, naked mole rats, ocean quahog, rockfish, and Greenland sharks, have shown negligible senescence or no signs of aging [23,24,25,26]. Naked mole rats, for example, are long-lived and do not show an accelerated mortality rate, which suggests the possibility of slowed or stopped aging [27,28]. However, the concept of negligible senescence has been criticized for underestimating mortality risk due to methodological issues [26].

Secondly, a growing number of discussions have been about reprogramming epigenetic changes as a potential way to reverse cellular aging [29,30,31,32]. Senescent cells show telomere shortening, DNA damage, modifications, epigenetic changes in transcription, and loss of epigenetic information, among others [33,34]. Interestingly, cellular senescence induced by passaging multiple times (Hayflick limit) or other methods can be converted to show a genome-wide transcript profile similar to the young cells through chemical cocktails or induced pluripotent stem cell (iPSC) reprogramming [30,35,36,37]. Macromolecular damage and modifications, including DNA damage in the nuclei and mitochondria, are major challenges in cell reprogramming, in which DNA repair and stress resistance play a critical role in cells from older ages. The details of potential challenges have been discussed elsewhere [37,38].

To translate the gene information and bridge the knowledge gap in humans, we investigated the biological pathways related to stress resistance in humans. We extracted a list of genes associated with stress resistance in humans and investigated biological pathways with a medium confidence level (*p*-value < 1 × 10^−4^). Our findings revealed that pathways associated with stress resistance overlapped with most of the longevity pathways previously identified [39]. However, the remaining categories did not overlap with longevity.

## 2. Results

### 2.1. Stress Resistance

#### 2.1.1. Databases and Overview of Stress Resistance

Using more than 180 databases, we raked human genes relevant to stress resistance. We used two comparisons to assess various enrichment methods. Firstly, we compared gene enrichment dimensions (Table 1). Although the biological process had the highest number of hits (1350 terms), the Reactome pathways had the highest number of pathway hits (468 pathways), making the Reactome more suitable for gene network analysis. Secondly, we compared 12 databases compatible with gene set enrichment analysis and found the following number of pathways identified by stress-resistance genes with “high” confidence of *p*-value ≤ 0.0001 (out of the total number of pathways in the database): Reactome 122 (out of 143); PubChem 54 (out of 65); GeneGo 51 (out of 58); Qiagen 58 (out of 59); R&D Systems 12 (out of 12); PharmGKB 9 (out of 10); and Cell Signaling Technology 8 (out of 9). We chose Reactome as it provided the highest number of pathways (accessed on 17 September 2024). It is worth noting that Sino Biological, Tocris Bioscience, GeneTex, BosterBio, and MedChemExpress did not identify pathways using the program Genecards. Additionally, the program extracts pathways from 13 resources, excluding wiki pathways, resulting in 12 resources.

**Figure 1:**
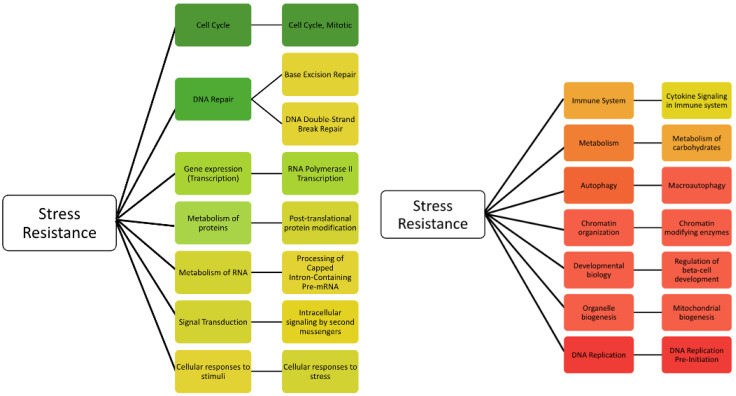
The 14 general pathways linked to the stress-resistance genes (Table S1). They are color-coded to the number of occurrences of the pathway. Green represents the general pathway with the most hits and red represents the general pathway with the least hits. To the right of the general pathways are the specific pathways. The specific pathways are color-coded to the number of occurrences of the pathway in relation to the other most frequent specific pathways.

Using the databases, we raked human genes relevant to stress resistance and identified 541 genes associated with the term stress resistance using Genecards. The validation of the gene set was performed to run the gene set using another program, STRING-DB. Two pathway analyses showed categories relevant to stress resistance with the highest significance among all of the pathways, the Reactome (FDR = 3.12 × 10^−51^) and the STRING-DB Biological process (FDR = 4.08 × 10^−59^). The results suggest that the gene set is enriched with genes related to stress resistance. We found diverse ontology nomenclatures, including Biological, Molecular, Cellular, KEGG, and Reactome dimensions, among others, and thus we decided to use the Reactome analysis for consistency with our previous studies [39,40]. The gene set enrichment analysis for the 541 stress-resistance genes compiled 398 biological Reactome pathways. Of them, 172 biological pathways met the threshold of a *p*-value < 1 × 10^−4^. The 172 biological pathways were summarized into 14 general pathways. The general pathways, in the order of most frequent to least frequent, are cell cycle, DNA repair, gene expression (transcription), metabolism of proteins, metabolism of RNA, signal transduction, cellular responses to stimuli, immune system, metabolism, autophagy, chromatin organization, developmental biology, organelle biogenesis, and DNA replication. Figure 1 summarizes the identified 14 general pathways and the sub-pathways with the highest number of hits for each general pathway.

#### 2.1.2. Stress Resistance: Cell-Cycle-Related Pathways

Table 2 summarizes the results for the selected stress-resistance biological pathways with the general pathway category cell cycle. Table 3 covers top hits and Table S1 covers all of the 172 pathways. The cell-cycle-related category was composed of four general pathways, including cell cycle, DNA repair, DNA replication, and chromatin organization; they are related to the functions of the cell cycle (Table 2). The cell cycle preceded specific pathways in the order of the most frequent: M-phase, S-phase, and checkpoints (G1/S and mitotic spindle checkpoints). Notably, the M-phase pathways included specific phases of M-phase (prophase, metaphase, telophase, among others), sister chromatid cohesion and separation, meiosis, and nuclear envelope/lamina, including, for example, the Hutchinson–Gilford progeria syndrome (HGPS) gene (LMNA). Six of the cell cycle pathways included telomere maintenance and DNA synthesis, lagging and leading strands, and the establishment of sister chromatid cohesion. The cellular response to stimuli included general response to stress, response to heat stress and HSF-1 regulations, and cellular senescence dependent on and independent of oxidative damage. The pathway category of chromatin organization included histone and modifiers, HDAC histone deacetylases, HDACs, and the NAD-dependent deacetylase sirtuin-1, SIRT1, which is involved in diverse functions of epigenetic regulation [41,42].

The second most frequent hit of the 14 general pathways was within the category of DNA repair. DNA repair is known to link to cell cycle functions, including DNA replication and checkpoint. The most frequent specific pathways for DNA repair covered most if not all repair systems, including base excision repair and DNA double-strand break repair, each with nine hits, while others included DNA damage bypass (eight pathways), mismatch repair (three pathways), and nucleotide excision repair (eight pathways). Importantly, the pathways included another progeria gene, the Werner syndrome gene (WRN), which is a RecQ-like DNA helicase gene. WRN was involved in the cell cycle, such as cell cycle checkpoints and telomere maintenance; in the DNA-related pathways, such as homology-directed repair and double-strand break repair; and the gene expression.

#### 2.1.3. Stress Resistance: Signal Transduction–Gene Expression Pathways

The signal-transduction-related pathways included two major pathway categories, signal transduction and gene expression. The signal transduction pathways identified 14 pathways composed of intracellular signaling by second messengers and other signaling pathways. They included stress-resistance pathways, which can be divided into several known pathways mediated by insulin-PIP3-AKT-FOXO, NOTCH, nuclear receptors (ESR-mediated estrogen-dependent gene expression), Rho GTPases, and WNT-beta-catenin). Importantly, mTOR was involved in and scattered among 13 pathways ranging from macroautophagy (one pathway), cellular stress response (four pathways), RNA Polymerase II Transcription (two pathways), regulation of p53 (two pathways), and PIP3/PTEN-AKT pathways (four pathways).

The gene expression pathways were identified as 19 pathways, including RNA polymerase II transcription (15 specific pathways), epigenetic regulation of gene expression (2 specific pathways), and gene silencing by RNA (2 specific pathways). RNA polymerase II transcription included the notable genes TP53, FOXO (FOXO1,3,4), RUNX2, VENTX, Notch-HLH, and p14^ARF^, among others. Epigenetic regulation of gene expression included ERCC6, EHMT2, HDACs (HDAC1 and 2), and histone genes (H2s and H4C1). Gene silencing by RNA included a total of 25 genes, including mostly NUPs and histone genes (H2AZ2 and H4C1). Interestingly, SIRTs (SIRT1,3), known as gene-silencing genes, were identified as genes in gene expression but not in the epigenetic regulation of gene expression.

#### 2.1.4. Stress Resistance: Metabolic Pathways

The metabolic pathways included the metabolism of carbohydrates, the metabolism of non-coding RNA, and organelle biogenesis and maintenance, according to the Reactome categories. The categories metabolism of proteins and metabolism of RNA are described in Section 2.1.5. Of 172 pathways, 4 pathways fell into the metabolism of carbohydrates, while 2 pathways belong to organelle biogenesis and maintenance in the mitochondria (Table S1). Interestingly, the metabolism of carbohydrates included mostly proteins in the transport and regulation of metabolic enzymes, including the nuclear pore proteins, nucleoporin (NUP) genes (16 genes), and glucokinase, which involves transport systems between the cytoplasm and the nucleus. Although the vast majority of the genes in this group fell into the regulatory genes of metabolism, they were mostly not in the metabolic enzymes. Thus, this category represented the transport and regulation of them rather than the metabolic enzymes themselves. Importantly, the nuclear pore proteins (NUPs) were enriched in 46 biological pathways relevant to the transport of macromolecules and nuclear envelope assembly; they were involved in glucose and carbohydrate metabolisms (4 pathways), pre-matured and matured RNA (11 pathways), SUMOylation-dependent protein degradation (9 pathways), cellular response to heat (2 pathways), interferon anti-virus immune system (3 pathways), cell cycle (14 pathways), and gene silencing (2 pathways), among others. This result highlights the role of the nuclear envelope’s integrity and the transport of macromolecules in the biological pathways for stress resistance. This result also suggests that the category of metabolism may not represent metabolic enzymes but mostly proteins in the transport and regulation of metabolism. Importantly, the accelerated aging syndrome (progeria) gene, HGPS gene (LMNA), is also included in this group related to the structure of the nuclear envelope and pores.

#### 2.1.5. Stress Resistance: Metabolism of Proteins, Metabolism of RNA, and Others

Metabolism of proteins: of 172 pathways, 11 pathways were identified in the category metabolism of proteins, all of which were proteins involved in protein degradation called SUMOylation. They include the SUMOylation of ubiquitinated proteins, chromatin organization proteins, DNA damage response and repair proteins, SUMOylation proteins, RNA-binding proteins, and DNA replication proteins. Thus, they overlapped with the cell cycle (DNA repair, replication, and chromatin organizations). As described above, 9 out of 11 pathways were enriched with the nuclear pore proteins (NUPs).

Other categories were as follows. Mitochondrial biogenesis: two pathways were identified as the mitochondrial biogenesis pathways, which were part of energy metabolism. Metabolism of RNA: nine pathways were identified as the metabolism of RNA pathways. They were involved in the processing of capped intron-containing pre-mRNA, including the transport of mature mRNAs derived from transcripts (seven pathways) and mRNA splicing (two pathways). Cellular responses to stimuli: 11 pathways fell into this category, including heat response dependent on HSF-1 (5 pathways) and HSP90 chaperon (1 pathway), response to starvation (1pathway), response to starvation (1 pathway), response to amino acid deficiency (1 pathway), and cellular senescence (2 pathways), including senescence induced by oxidative stress, among others. Immune system: six pathways were included, including innate immunity (one pathway) and cytokine signaling (five pathways), such as interferon signaling (three pathways) and interleukin signaling (one pathway). Autophagy: three pathways included macroautophagy (two pathways) and chaperone-mediated autophagy (one pathway). Developmental biology: two pathways identified beta-cell development, which is essential for insulin secretion.

### 2.2. Longevity

**Figure 2:**
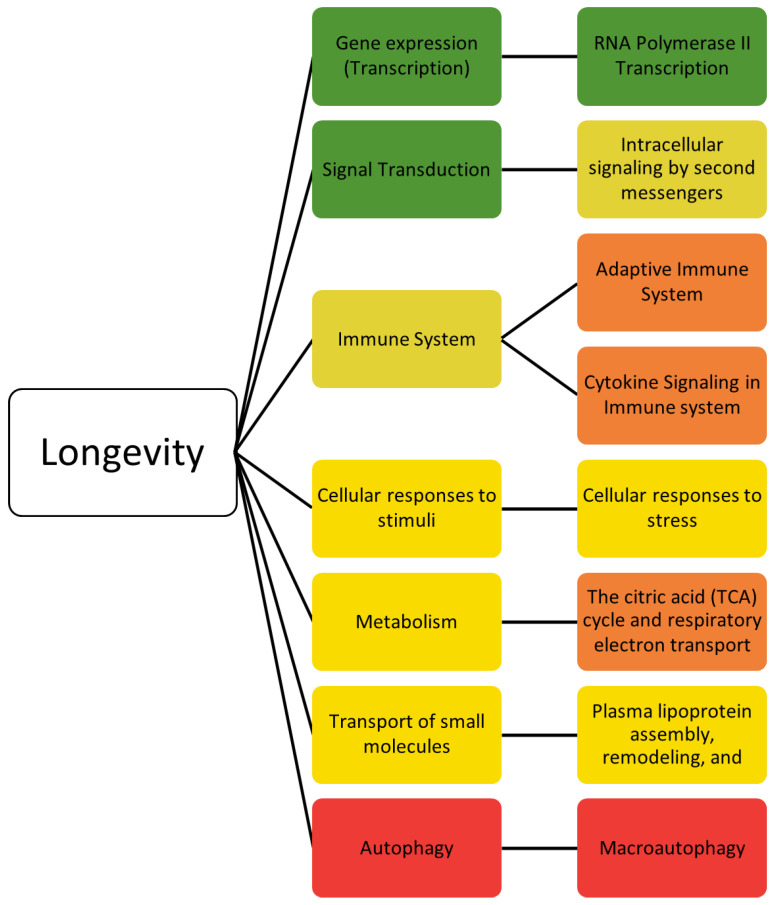
Seven general pathways associated with the longevity genes (Table S2). The color of the general pathway represents the number of occurrences of the pathway relative to the other general pathways. The pathways in green have the greatest number of hits, whereas the red have the fewest number of hits. To the right of the general pathways are the most frequent specific pathways. The specific pathways are color-coded in relation to the other most frequent specific pathways, with green being the most frequent and red being the least frequent.

The 357 longevity genes and the detailed pathways have been described previously [39]. Top-hit longevity pathways are shown in Table 4. In this study, we performed a more in-depth analysis of the pathways with a threshold of a *p*-value < 1 × 10^−4^. Firstly, the gene set enrichment analysis identified 141 biological pathways, narrowed down to 50 biological pathways with the *p*-value threshold. The 50 biological pathways were summarized into 7 general pathways. The seven general pathways, in the order of most frequent to least frequent result, are gene expression (transcription), signal transduction, immune system, cellular responses to stimuli, metabolism, transport of small molecules, and autophagy. Figure 2 summarizes the identified seven general pathways and the most frequent sub-pathways for each of the seven general pathways.

Out of 50 pathways, the highest number of hits fell into gene expression (transcription) and signal transduction, with 14 pathways each. As expected, the pathways including FOXO, which are known to affect lifespans in the model systems, are overlapped between gene expression and signal transduction, with 7 out of 14 pathways as gene expression (FOXO-mediated gene expression) and 5 pathways as signal transduction (PIP3/PTEN-AKT pathways). The seven remaining gene expression pathways fell into TP53 regulation (four pathways), AP-2 (TFAR2) regulation (two pathways), and RUNX3-dependent CDKN1A transcription (one pathway). Another hit among the more specific pathways within signal transduction was intracellular signaling by second messengers. Table 3 summarizes the results of the selected longevity biological pathways with gene expression (transcription) and signal transduction as the general pathways.

### 2.3. Overlap for Stress Resistance and Longevity

**Figure 3:**
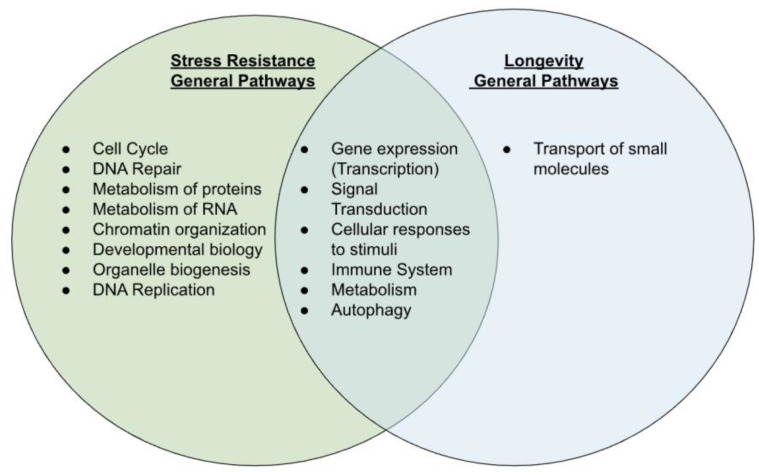
Compares and contrasts the general pathways linked to the stress-resistance genes and longevity genes. Eight general pathways are linked to only stress resistance, whereas one general pathway is linked to only longevity. Six general pathways are linked to both stress resistance and longevity.

**Figure 4:**
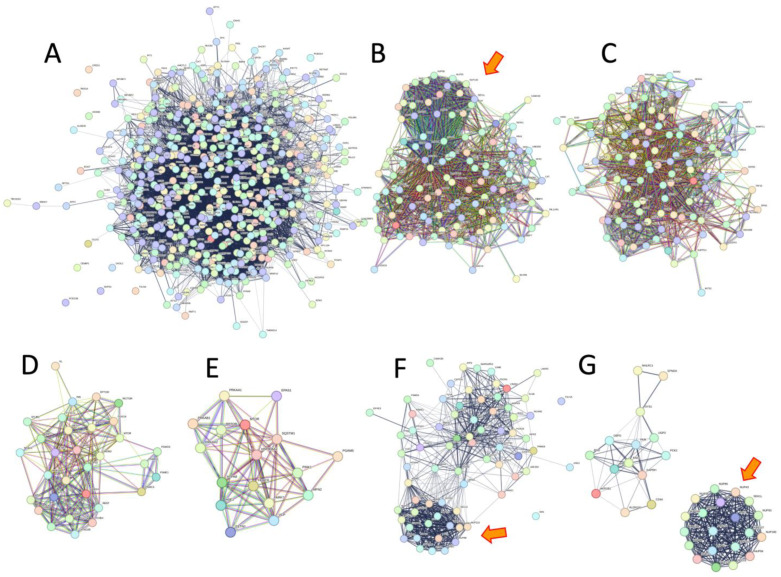
Interaction network diagrams of overlapped pathways. (**A**) Overall network of stress-resistance genes, (**B**) cellular response to stimuli (stress), (**C**) gene expression, (**D**) signal transduction, (**E**) autophagy, (**F**) immune system, (**G**) metabolism (carbohydrate). Red arrows indicate a cluster of the nuclear transport and integrity genes (NUPs), although the genes are embedded throughout the pathways, as described in the text. To generate these diagrams, we used the pathways with the highest number of hits in each category (Table S1).

Figure 3 summarizes biological pathway categories overlapped and non-overlapped between stress resistance and longevity; Figure 4 shows interaction network diagrams of overlapped pathways. Six pathway categories overlapped between the stress-resistance genes and the longevity genes. The overlapping general pathways were gene expression (transcription), signal transduction, cellular responses to stimuli, immune system, metabolism, and autophagy. Importantly, the overlapped categories include the PIP3-AKT-FOXO and mTOR pathways (categories gene expression and signal transduction) (Table S2), which are known to control life extension and/or stress resistance in the model systems. They also included the accelerated aging syndrome (progeria) genes, including the Hutchinson–Gilford progeria syndrome (HGPS) gene (LMNA) and the Werner syndrome gene (WRN) (categories cellular response to stimuli/stress), while the genes were also included in non-overlapped categories (DNA repair and cell cycle) (Table S2). The other longevity pathways included the transport of small molecules, which were involved in lipoprotein metabolism. Dysregulation of lipoprotein metabolism (i.e., oxidation and glycation) can cause atherosclerotic lesions, which, in broader classifications, may be considered stress damage. Interestingly, a cluster of the nuclear transport and integrity genes (NUPs) was observed in the interaction network in three pathway categories: cellular response to stimuli (stress), immune system, and metabolism (Figure 4). Other categories involving NUPs include signal transduction and gene expression in the overlapping categories (Table S1), while they include cell cycle, metabolisms of RNA, and proteins in the non-overlapping categories (Table S1). In summary, the longevity pathways mostly overlapped with stress-resistance pathways. In contrast, the other stress-resistance pathways not overlapped in longevity were specific to molecular (DNA and RNA) and biological categories (cell cycle, chromatin, organelle, and post-translational modification/metabolism of proteins) (Figure 3).

## 3. Discussion

Previous research on stress resistance focused on specific mechanisms in model systems, leading to a significant gap in humans due to biological differences. Thus, it has been challenging to translate findings into clinical therapeutic interventions due to biological barriers. In this study, we used gene set enrichment analysis (GSEA) to uncover the gene networks to explore the gene networks associated with stress resistance and longevity in humans. We successfully identified 541 genes linked to stress resistance, which is more comprehensive than the previously documented 215 single-nucleotide polymorphisms associated with longevity in individuals who smoke [43]. The results show that the genes are involved in 172 pathways. Moreover, we found that these pathways can be grouped into 14 major pathways, which are closely related to 6 out of the 7 longevity pathways previously obtained from 50 biological pathways.

### 3.1. Similarity among Stress Resistance and Longevity Pathways

Six pathway categories are common between stress resistance and longevity genes. The categories include gene expression (transcription), signal transduction, immune system, cellular responses to stimuli, metabolism, and autophagy. In-depth investigation identifies the well-known pathways for stress resistance and longevity in the model systems, such as the PIP3/PTEN-AKT-FOXO pathway, that are covered mainly under the gene expression and the signal transduction pathways. These pathways are regulatory pathways that govern other biological pathways. If life extension is the result of reducing the causes of aging, then stress resistance should be involved in the causes of aging (i.e., stress resistance related to longevity). Importantly, the common categories also include the progeria genes (HGPS/LMNA and WRN), while the genes are also included in non-overlapped categories.

The common pathway categories between stress resistance and longevity suggest underlying mechanisms. Stress resistance confers increased survival after exposure to oxidative stress, heat, UV light, DNA-damaging agents, toxic peptides, and other types of stress. Previous studies suggest that multiplex stress resistance unifies multiple forms of single-stress resistance and is essential for life extension [13,16]. It seems reasonable to state that a wide variety of forms of stress resistance is under the control of limited biological mechanisms, such as, for example, serotonin and insulin/IGF-1 pathways [19,20,21,22,44], as well as the genetic mechanisms for stress-resistance categories related to longevity. It is worth noting that stress resistance may be altered depending on the intrinsic stress conditions; for example, supplementing glucose metabolites (pyruvate and lactate) causes increases in oxidative stress and triggers hormesis (also called biological resilience) and life extension [45].

### 3.2. Differences among Stress Resistance and Longevity Pathways

In this study, a clear difference between stress resistance and longevity is that stress resistance covers broader pathways; in total, 172 stress-resistance pathways are summarized into 14 categories, while 141 pathways are summarized into 7 categories. Of the 14, 8 stress-resistance categories unique to stress are enriched in the functions related to cell proliferation and development, including cell cycle, DNA repair, metabolism of proteins, metabolism of RNA, chromatin organization, developmental biology (of beta-cell), organelle biogenesis (in the mitochondria), and DNA replication.

There are two major differences between experimental stress resistance and longevity. (1) The nature of stress damage: stress resistance assesses survival under exposure to a sublethal stressor, while such excess stress is not expected in normal aging. (2) Sites of stress damage: each form of excess stress has a specific site of damage in the whole body, while any stress is experimentally minimized in normal aging. Thus, single-stress resistance is segmental and may be insufficient for life extension. Resistance to a single stress (e.g., oxidative stress and heat) can be separated from life extension in worms [8,46]. Importantly, suppressing superoxide dismutase genes reduces life extension through a life-extending *daf-2* (Insulin/IGF-1 receptor gene) mutation [15,46], suggesting that single-stress resistance may still count for a part of life extension.

Notably, lipoprotein metabolism is included in longevity but not stress resistance in the Reactome-defined pathways. However, low-density lipoprotein (LDL) is involved in a part of lipoprotein metabolism, which is known to trigger atherosclerotic lesions through oxidation and glycation [47,48] and thus may be viewed as stress damage on LDL. Thus, we propose to include a Reactome subcategory of stress damage under the category of lipoprotein metabolism. In humans, the leading cause of death has been cardiovascular diseases [49,50]. This suggests specific parts of the body, such as the circulation systems, play a critical role in mortality.

### 3.3. Translation of the Results into Potential Therapeutic Targets or Interventions

#### 3.3.1. Overlapped Pathway Categories between Stress Resistance and Longevity

Several life-extending interventions have been under clinical trials, including rapamycin, metformin, and nicotinamide adenine dinucleotide precursors [51,52]. There are seven groups reported [51], which are well-translated into potential therapeutic interventions into our general categories (types of pharmacological interventions), including signal transduction (mTOR inhibitors and insulin pathway-related drugs, e.g., rapamycin, metformin, 17-alpha-estradiol, acarbose, and FGF21), autophagy (e.g., spermidine) and gene expression (NAD precursors, e.g., nicotinamide riboside), and metabolism (restriction of specific amino acids, e.g., protein restriction, methionine restriction, tryptophan restriction), among others. Notably, one therapeutic intervention (the category of metabolism of protein or amino acid/protein restriction) fits better in non-overlapping pathways specific to stress resistance (see below).

Our findings (Figure 3) are also consistent with the notion that life-extending interventions can be grouped into a few mechanisms, including nutrient signaling, mitochondrial proteostasis, and the autophagic machinery [53], but they also point to broader categories. The signal transduction and gene expression categories include the PIP3/PTEN-AKT-FOXO pathway relevant to nutrient sensing; mitochondrial proteostasis was embedded in metabolism (Section 2.1.4). Autophagy showed up as a stand-alone category in overlapped pathways. Additionally, the overlapped categories point out cellular responses to stimuli, the immune system, and other aspects of metabolism.

#### 3.3.2. Non-Overlapped Pathway Categories between Stress Resistance and Longevity

The pathways specific to stress resistance can be grouped into three categories: cell division (including cell cycle, chromatin organization, and DNA replication), damage repair (DNA repair), and metabolism (involving the metabolism of proteins, RNA, and organelle biogenesis). Interestingly, the metabolism category, particularly the metabolism of proteins, may be translated into a type of life-extending intervention being tested in humans (protein restriction, methionine restriction, and tryptophan restriction). Furthermore, non-overlapping categories are mostly clustered among cell and tissue maintenance categories, including cell cycle, DNA repair, DNA replication, chromatin organization, and organelle biogenesis, among others (Figure 3), which have direct links to epigenetics and its reprogramming to promote cell proliferation. Consistently, cellular senescence and epigenetic regulations are found under the non-overlapping part of cellular response to stimuli (stress) and gene expression (Table S1). Our findings (Figure 3) indicate non-overlapping categories that trend towards cell and tissue maintenance, potentially linked to age-related changes rather than life extension.

### 3.4. Advantages and Limitations of the Method

The advantages and limitations of the Reactome analysis have been discussed previously [39]. Human phenotypes primarily result from multiple polymorphisms and multifactorial differences, including genetic, phenotypic, and clinical heterogeneity among others [39,40,54,55,56,57]. The gene-network analysis, such as GSEA, effectively explores conditions in humans in which single-gene mutational approaches are not impractical. Visualizing the genetic network based on the pathway categories provides insights that may have been missed previously. Considering that most gene effects in humans are epistatic, quantitative, or multifactorial, gene network analysis is a valuable means of providing an overview of the genetic effects in humans.

The limitations of GSEA are as follows. Firstly, the list of the genes generated is a living knowledge that needs to be updated periodically. Secondly, the analysis outputs are multi-dimensional ontology pathways organized by each dimension, ranging from molecules, cellular/extracellular, tissue, functional, and phenotypic, among others, where nomenclature is different to indicate similar biological phenomena. For example, we found the pathway category cellular response to cells in the Reactome analysis and STRING-DB biological pathways; however, other pathway analyses do not allow for straightforward identification of the categories related to stress resistance.

Moreover, we had difficulty with terminology aligning with the aging hallmarks previously reported [58], which are defined as a mixture of various ontology dimensions ranging from biological to other categories. For example, categories related to nutrition (deregulated nutrient-sensing), stem cells (stem cell exhaustion), and telomeres (telomere attrition) are at the levels of the macromolecule, the cell, and the chromosomal domain, which are at different ontology dimensions. It is also not clear how the aging hallmarks fit in well-established pathways for longevity and stress resistance, including the Insulin/IGF-1 pathways, whose second messengers are PIP3-AKT-FOXO, and the mTOR pathways. To minimize discrepancies and to have more consistency, we have used the Reactome analysis, which has been relatively accepted and incorporated into various network analyses. Finally, due to the reason above, the aging hallmark terminologies do not align with the ontology pathway categories with various dimensions. We propose to have more precise biological terminologies defining multi-dimensional nomenclature, including molecular, cellular/extracellular, tissue, and systemic levels, among others, to provide more unified and integral knowledge as new findings keep coming into this field.

### 3.5. Nuclear Transport and Integrity Emphasized Stress Resistance and Aging but Not Longevity

We found that nuclear pore proteins (NUPs) are enriched in 46 biological pathways for stress resistance (Table S1). NUPs are relevant to the transportation of macromolecules and the assembly of the nuclear envelope. NUPs are the main components of the nuclear pore complexes. They are associated with scaffold complexes, including the NUP107-160 complex and NUP93-205 complex, the NUP214 complex, the NUP98 complex, and the NUP62 complex, among others [59,60]. The NUP62, 93, and 152 play a role in nuclear transport, which is suggested to be involved in aging [60]. Additionally, nuclear envelope integrity has been proposed to play a role in aging. Firstly, the Hutchinson–Gilford progeria syndrome (HGPS), also known as accelerated aging syndrome or progeria, is caused by mutations in the LMNA gene that encodes Lamin A/C [61], and it is tightly related to the structure of the nuclear envelope and the pores [60,62]. In this study, the NUPs are involved in diverse pathways, including carbohydrate metabolism, RNA and proteins, the cellular stress response, the anti-virus immune system, the cell cycle, and gene silencing, among others. However, the NUPs are not involved in longevity pathways. We suggest that NUP-dependent nuclear integrity and transport play a role in aging and stress resistance but not longevity. Taken together, the results suggest a novel role of NUP-dependent nuclear integrity and transport in age-related metabolic changes and diverse pathways.

## 4. Materials and Methods

### 4.1. Nomenclatures

We define “single stress resistance” as the ability of an organism to withstand a specific type of stress, such as oxidative stress, heat, or chemical exposure, and “multiplex stress resistance”, on the other hand, as the ability of an organism to withstand multiple forms of stress. To avoid complex ontology nomenclatures, we used the terms as previously described [39,40]. Briefly, the Reactome ontology pathways are composed of two or more levels, ranging from the most general pathways (referred to as pathway categories or, simply, categories) divided into more specific pathways to specific pathways (referred to as specific pathways or simply pathways), among others. For example, a pathway category of autophagy has in the order of autophagy (pathway category) > macroautophagy (more specific pathway) > selective autophagy (specific pathway). We have grouped all ontology pathways into each general pathway, namely, pathway categories.

### 4.2. Datasets

A pool of the curated gene set was initially identified using Genecards.org; it is a bioinformatic tool that ciphers through more than 180 scientific databases by inputting a search query and providing a list associated with human genes [63] “https://www.genecards.org/ (last accessed on 17 March 2023; Table S3)”. The genes relevant to stress resistance were searched using the platform Elastic search 7.11 (https://www.genecards.org/Guide/Search#relevance) (last accessed on 17 March 2023), which is based on the Boolean model to retrieve matching documents and the relevance score calculated using Lucene’s practical scoring function. The base keywords “stress resistance” AND “humans” were used to search the genes with the relevance score. The scores are categorized into three quality levels based on the *p*-value, as follows [64,65]: high (*p*-value ≤ 0.0001); medium (*p*-value > 0.0001 but ≤0.05); and low (*p*-value > 0.05). We used the high and medium confidence levels to extract the gene set. We further validated the relevance to stress resistance using Reactome and STRING-DB [66], as described in the text and also as described previously [39]. Reactome calculates confidence based on two types of confidence levels (*p*-value and false detection rate), as described previously [67]. STRING-DB shows a combined score, which is calculated by aggregating the probabilities from different evidence channels and adjusting for the likelihood of randomly observing an interaction [66].

### 4.3. Gene Ontology

The Reactome analysis and STRING-DB analysis were performed as described previously [39,40]. The Cytoscape plugin Reactome FIViz classified and assembled the exported gene list into biological pathways. Reactome utilizes a systematic accumulation of pathway databases that performs analyses of biological pathways. The biological pathways are organized in a stratified fashion, often with sub-pathways. Sub-pathways are strings linked to an arching pathway; therefore, hit genes can overlap with different arching pathways. The exported gene list associated with stress resistance was inputted into the Reactome FIViz program. The program then executed and carried out a range of gene set analyses at a False Discovery Rate (FDR) of 0.05. The research team independently conducted the task to cross-reference and validate the retrieved gene set analysis. We used more stringent conditions at a threshold *p*-value of <1 × 10^−4^. which is roughly comparable to the FDR 1 × 10^−4^. The retrieved gene set analysis was categorically arranged to eliminate redundancies. Categorization, as previously described [39,40], was based on biological mapping pathways with a gamut from general pathways to specific pathways. For example, a general hallmark would be the immune pathway, where a more specific pathway would be cytokine signaling in immune systems, and a specific pathway would be interferon signaling. With regard to longevity gene data, a prior biological pathway list has been described [39,40]. Two independent individuals randomly collected, performed, and verified the analysis of the categorization.

## 5. Conclusions

This study indicates that the biological pathways responsible for longevity are closely linked with those involved in stress resistance. This finding suggests that the pathways that play a role in longevity are also important for stress resistance, which aligns with previous studies on multiplex stress resistance [5,16,17,18]. We found that longevity pathways were summarized into six categories. Assuming that suppressing the causes of aging should delay aging and lead to life extension, the causes of aging may be limited. Stress resistance related to longevity should be involved in the causes of aging, while stress resistance unrelated to longevity may be the consequence of aging and responding to stress damage. Similarly, progeria genes are involved in DNA and nuclear integrity [68,69], which may be involved in both the cause and consequences of aging. The findings in this study appear to support initial age-related causes transitioning to more complex consequences of aging with a mixture of aging physiology and pathology. Previous studies suggest that there is a transitional phase before advanced aging; the transitional phase formulated the ground of the aging theory (middle-life crisis theory) [20,21,22].

This study shows that multiplex stress resistance involves mechanisms unifying responses and resistances to multiple forms of stress. In addition, stress resistance is likely not limited to molecular damage but also extends to lipid/lipoprotein metabolism that includes the oxidation and glycation of LDL, which is not currently classified as damage in the Reactome ontology analysis. This study contributes a critical step forward in the fight against aging and age-related diseases. This study reinstates the significance of stress resistance and longevity, while it supports distinct mechanisms of aging and longevity.

## Figures and Tables

**Table 1:** Number of hits in each functional enrichment dimension.

Functional Enrichment Dimensions	Number of Hits
Biological Process (Gene Ontology)	1359 GO-terms
Molecular Function (Gene Ontology)	162 GO-terms
Cellular Component (Gene Ontology)	163 GO-terms
Reference publications (PubMed)	10,000 publications
Local network cluster (STRING)	116 clusters
KEGG Pathways	125 pathways
Reactome Pathways	468 pathways
GO (gene ontology)	

**Table 2:** Summary of stress-resistance pathway categories identified by Reactome and breakdowns.

Pathways	No. of Pathways
Total Number of Pathways Identified	172
Total Number of Pathway Categories Grouped *	14
**Breakdowns of 172 Pathway Categories**	No. of pathways
1 Cell Cycle	40
2 DNA Repair	37
3 Gene Expression (Transcription)	19
4 Metabolism of Proteins	17
5 Metabolism of RNA	14
6 Signal Transduction	14
7 Cellular Responses to Stimuli	11
8 Immune System	6
9 Metabolism	4
10 Autophagy	3
11 Chromatin Organization	2
12 Developmental Biology	2
13 Organelle Biogenesis	2
14 DNA Replication	1
**Total**	**172**

*, The grouped categories are shown with a number.

**Table 3:** Top 27 stress-resistance pathways. The general, specific, and more specific pathways were organized and listed for each of the 27 Reactome pathways. All of the pathways in Table 2 fall under the cell cycle as the overarching pathway. The ratio of protein in the pathway, the number of proteins in the pathway, the protein from the gene set, the *p*-value, the FDR, and hit genes were provided by executing a gene set enrichment analysis.

Selected Pathways for Stress-Resistance Pathway Enrichment									
Reactome Pathway	Ratio of Protein in Pathway	Number of Protein in Pathway	Protein from Gene Set	*p*-Value	FDR	Hit Genes	General	Specific	More Specific
Cell Cycle, Mitotic	0.0478	530	86	1.11 × 10^−16^	4.44 × 10^−15^	*NUP107*,*SMC3*,*SMC4*,*SMC2*,*MYC*,*AKT1*,*NUP210*,*PRKCA*,*NUP93*,*PSME1*,*NUP98*,*NUP205*,*SEH1L*,*NUP85*,*RAD21*,*NUP88*,*PLK1*,*TUBA1B*,*TUBA1A*,*EMD*,*NDC1*,*SIRT2*,*TUBB2A*,*TUBB4B*,*PSMC6*,*PSMC3*,*NCAPD2*,*ANAPC7*,*LEMD2*,*KPNB1*,*ANAPC1*,*HSP90AB1*,*MCM7*,*FOXM1*,*TNPO1*,*RFC5*,*RFC3*,*HSP90AA1*,*RFC4*,*RFC1*,*RFC2*,*RANGAP1*,*SMC1A*,*MCM6*,*MCM2*,*HDAC1*,*RBBP4*,*H2AZ2*,*CDC27*,*RCC1*,*H4C1*,*SKP2*,*TMPO*,*TP53*,*WAPL*,*TOP2A*,*CETN2*,*SEC13*,*TUBB*,*SFI1*,*PCNA*,*LMNA*,*RAE1*,*H2BC3*,*RPA1*,*RPA2*,*RPA3*,*NUP188*,*CCND1*,*BANF1*,*PSMD11*,*PDS5A*,*NUP160*,*UBE2I*,*NUP155*,*LMNB1*,*NUP62*,*NUP133*,*NUP58*,*POLD3*,*POLD2*,*NUP43*,*BUB3*,*NUP35*,*RAN*,*NUP37*	Cell Cycle	Cell Cycle, Mitotic	Cell Cycle, Mitotic
Mitotic Prophase	0.0101	112	33	1.11 × 10^−16^	4.44 × 10^−15^	*NUP107*,*SMC4*,*SMC2*,*NUP210*,*PRKCA*,*NUP93*,*NUP98*,*NUP205*,*SEH1L*,*NUP85*,*NUP88*,*PLK1*,*EMD*,*NDC1*,*LEMD2*,*H2AZ2*,*H4C1*,*TMPO*,*SEC13*,*LMNA*,*RAE1*,*H2BC3*,*NUP188*,*BANF1*,*NUP160*,*NUP155*,*LMNB1*,*NUP62*,*NUP133*,*NUP58*,*NUP43*,*NUP35*,*NUP37*	Cell Cycle	Cell Cycle, Mitotic	Mitotic Prophase
Mitotic Anaphase	0.0212	235	48	1.11 × 10^−16^	4.44 × 10^−15^	*NUP107*,*SMC3*,*NUP93*,*PSME1*,*NUP98*,*NUP205*,*SEH1L*,*NUP85*,*RAD21*,*PLK1*,*TUBA1B*,*TUBA1A*,*EMD*,*NDC1*,*SIRT2*,*TUBB2A*,*TUBB4B*,*PSMC6*,*PSMC3*,*ANAPC7*,*LEMD2*,*KPNB1*,*ANAPC1*,*TNPO1*,*RANGAP1*,*SMC1A*,*CDC27*,*RCC1*,*TMPO*,*WAPL*,*SEC13*,*LMNA*,*NUP188*,*BANF1*,*PSMD11*,*PDS5A*,*NUP160*,*UBE2I*,*NUP155*,*LMNB1*,*NUP62*,*NUP133*,*NUP58*,*NUP43*,*BUB3*,*NUP35*,*RAN*,*NUP37*	Cell Cycle	Cell Cycle, Mitotic	Mitotic Anaphase
Nuclear Envelope Breakdown	0.0048	53	28	1.11 × 10^−16^	4.44 × 10^−15^	*NUP107*,*NUP210*,*PRKCA*,*NUP93*,*NUP98*,*NUP205*,*SEH1L*,*NUP85*,*NUP88*,*PLK1*,*EMD*,*NDC1*,*LEMD2*,*TMPO*,*SEC13*,*LMNA*,*RAE1*,*NUP188*,*BANF1*,*NUP160*,*NUP155*,*LMNB1*,*NUP62*,*NUP133*,*NUP58*,*NUP43*,*NUP35*,*NUP37*	Cell Cycle	Cell Cycle, Mitotic	Nuclear Envelope Breakdown
M Phase	0.0348	386	62	1.11 × 10^−16^	4.44 × 10^−15^	*NUP107*,*SMC3*,*SMC4*,*SMC2*,*NUP210*,*PRKCA*,*NUP93*,*PSME1*,*NUP98*,*NUP205*,*SEH1L*,*NUP85*,*RAD21*,*NUP88*,*PLK1*,*TUBA1B*,*TUBA1A*,*EMD*,*NDC1*,*SIRT2*,*TUBB2A*,*TUBB4B*,*PSMC6*,*PSMC3*,*NCAPD2*,*ANAPC7*,*LEMD2*,*KPNB1*,*ANAPC1*,*TNPO1*,*HSP90AA1*,*RANGAP1*,*SMC1A*,*H2AZ2*,*CDC27*,*RCC1*,*H4C1*,*TMPO*,*WAPL*,*CETN2*,*SEC13*,*TUBB*,*SFI1*,*LMNA*,*RAE1*,*H2BC3*,*NUP188*,*BANF1*,*PSMD11*,*PDS5A*,*NUP160*,*UBE2I*,*NUP155*,*LMNB1*,*NUP62*,*NUP133*,*NUP58*,*NUP43*,*BUB3*,*NUP35*,*RAN*,*NUP37*	Cell Cycle	Cell Cycle, Mitotic	M Phase
Nuclear Pore Complex (NPC) Disassembly	0.0032	36	20	1.11 × 10^−16^	4.44 × 10^−15^	*NUP107*,*NUP210*,*NUP93*,*NUP98*,*NUP205*,*SEH1L*,*NUP85*,*NUP88*,*NDC1*,*SEC13*,*RAE1*,*NUP188*,*NUP160*,*NUP155*,*NUP62*,*NUP133*,*NUP58*,*NUP43*,*NUP35*,*NUP37*	Cell Cycle	Cell Cycle, Mitotic	Nuclear Pore Complex (NPC) Disassembly
Mitotic Metaphase and Anaphase	0.0213	236	48	1.11 × 10^−16^	4.44 × 10^−15^	*NUP107*,*SMC3*,*NUP93*,*PSME1*,*NUP98*,*NUP205*,*SEH1L*,*NUP85*,*RAD21*,*PLK1*,*TUBA1B*,*TUBA1A*,*EMD*,*NDC1*,*SIRT2*,*TUBB2A*,*TUBB4B*,*PSMC6*,*PSMC3*,*ANAPC7*,*LEMD2*,*KPNB1*,*ANAPC1*,*TNPO1*,*RANGAP1*,*SMC1A*,*CDC27*,*RCC1*,*TMPO*,*WAPL*,*SEC13*,*LMNA*,*NUP188*,*BANF1*,*PSMD11*,*PDS5A*,*NUP160*,*UBE2I*,*NUP155*,*LMNB1*,*NUP62*,*NUP133*,*NUP58*,*NUP43*,*BUB3*,*NUP35*,*RAN*,*NUP37*	Cell Cycle	Cell Cycle, Mitotic	Mitotic Metaphase and Anaphase
Postmitotic Nuclear Pore Complex (NPC) Reformation	0.0024	27	23	1.11 × 10^−16^	4.44 × 10^−15^	*NUP107*,*NUP93*,*NUP98*,*NUP205*,*SEH1L*,*NUP85*,*NDC1*,*KPNB1*,*TNPO1*,*RANGAP1*,*RCC1*,*SEC13*,*NUP188*,*NUP160*,*UBE2I*,*NUP155*,*NUP62*,*NUP133*,*NUP58*,*NUP43*,*NUP35*,*RAN*,*NUP37*	Cell Cycle	Cell Cycle, Mitotic	Postmitotic Nuclear Pore Complex (NPC) Reformation
Nuclear Envelope (NE) Reassembly	0.0068	76	34	1.11 × 10^−16^	4.44 × 10^−15^	*NUP107*,*NUP93*,*NUP98*,*NUP205*,*SEH1L*,*NUP85*,*TUBA1B*,*TUBA1A*,*EMD*,*NDC1*,*SIRT2*,*TUBB2A*,*TUBB4B*,*LEMD2*,*KPNB1*,*TNPO1*,*RANGAP1*,*RCC1*,*TMPO*,*SEC13*,*LMNA*,*NUP188*,*BANF1*,*NUP160*,*UBE2I*,*NUP155*,*LMNB1*,*NUP62*,*NUP133*,*NUP58*,*NUP43*,*NUP35*,*RAN*,*NUP37*	Cell Cycle	Cell Cycle, Mitotic	Nuclear Envelope (NE) Reassembly
S Phase	0.0147	163	31	2.68 × 10^−12^	6.43 × 10^−11^	*SMC3*,*MYC*,*AKT1*,*PSME1*,*RAD21*,*PSMC6*,*PSMC3*,*ANAPC7*,*ANAPC1*,*MCM7*,*RFC5*,*RFC3*,*RFC4*,*RFC1*,*RFC2*,*SMC1A*,*MCM6*,*MCM2*,*RBBP4*,*CDC27*,*SKP2*,*WAPL*,*PCNA*,*RPA1*,*RPA2*,*RPA3*,*CCND1*,*PSMD11*,*PDS5A*,*POLD3*,*POLD2*	Cell Cycle	Cell Cycle, Mitotic	S Phase
DNA Strand Elongation	0.0029	32	14	1.08 × 10^−10^	2.38 × 10^−9^	*MCM7*,*RFC5*,*RFC3*,*RFC4*,*RFC1*,*RFC2*,*MCM6*,*MCM2*,*PCNA*,*RPA1*,*RPA2*,*RPA3*,*POLD3*,*POLD2*	Cell Cycle	Cell Cycle, Mitotic	DNA Strand Elongation
Lagging Strand Synthesis	0.0018	20	11	1.05 × 10^−9^	1.99 × 10^−8^	*RFC5*,*RFC3*,*RFC4*,*RFC1*,*RFC2*,*PCNA*,*RPA1*,*RPA2*,*RPA3*,*POLD3*,*POLD2*	Cell Cycle	Cell Cycle, Mitotic	Lagging Strand Synthesis
Synthesis of DNA	0.0109	121	22	9.19 × 10^−9^	1.38 × 10^−7^	*PSME1*,*PSMC6*,*PSMC3*,*ANAPC7*,*ANAPC1*,*MCM7*,*RFC5*,*RFC3*,*RFC4*,*RFC1*,*RFC2*,*MCM6*,*MCM2*,*CDC27*,*SKP2*,*PCNA*,*RPA1*,*RPA2*,*RPA3*,*PSMD11*,*POLD3*,*POLD2*	Cell Cycle	Cell Cycle, Mitotic	Synthesis of DNA
Mitotic Prometaphase	0.0169	188	26	9.57 × 10^−8^	1.15 × 10^−6^	*NUP107*,*SMC3*,*SMC4*,*SMC2*,*NUP98*,*SEH1L*,*NUP85*,*RAD21*,*PLK1*,*TUBA1A*,*TUBB4B*,*NCAPD2*,*HSP90AA1*,*RANGAP1*,*SMC1A*,*WAPL*,*CETN2*,*SEC13*,*TUBB*,*SFI1*,*PDS5A*,*NUP160*,*NUP133*,*NUP43*,*BUB3*,*NUP37*	Cell Cycle	Cell Cycle, Mitotic	Mitotic Prometaphase
Polymerase Switching	0.0013	14	8	1.51 × 10^−7^	1.81 × 10^−6^	*RFC5*,*RFC3*,*RFC4*,*RFC1*,*RFC2*,*PCNA*,*POLD3*,*POLD2*	Cell Cycle	Cell Cycle, Mitotic	Polymerase switching
Leading Strand Synthesis	0.0013	14	8	1.51 × 10^−7^	1.81 × 10^−6^	*RFC5*,*RFC3*,*RFC4*,*RFC1*,*RFC2*,*PCNA*,*POLD3*,*POLD2*	Cell Cycle	Cell Cycle, Mitotic	Leading Strand Synthesis
Separation of Sister Chromatids	0.0155	172	24	2.55 × 10^−7^	2.81 × 10^−6^	*NUP107*,*SMC3*,*PSME1*,*NUP98*,*SEH1L*,*NUP85*,*RAD21*,*PLK1*,*PSMC6*,*PSMC3*,*ANAPC7*,*ANAPC1*,*RANGAP1*,*SMC1A*,*CDC27*,*WAPL*,*SEC13*,*PSMD11*,*PDS5A*,*NUP160*,*NUP133*,*NUP43*,*BUB3*,*NUP37*	Cell Cycle	Cell Cycle, Mitotic	Separation of Sister Chromatids
Initiation of Nuclear Envelope (NE) Reformation	0.0017	19	8	1.46 × 10^−6^	1.39 × 10^−5^	*EMD*,*SIRT2*,*LEMD2*,*KPNB1*,*TMPO*,*LMNA*,*BANF1*,*LMNB1*	Cell Cycle	Cell Cycle, Mitotic	Initiation of Nuclear Envelope (NE) Reformation
Resolution of Sister Chromatid Cohesion	0.0096	107	17	2.70 × 10^−6^	2.43 × 10^−5^	*NUP107*,*SMC3*,*NUP98*,*SEH1L*,*NUP85*,*RAD21*,*PLK1*,*RANGAP1*,*SMC1A*,*WAPL*,*SEC13*,*PDS5A*,*NUP160*,*NUP133*,*NUP43*,*BUB3*,*NUP37*	Cell Cycle	Cell Cycle, Mitotic	Resolution of Sister Chromatid Cohesion
Mitotic Telophase/Cytokinesis	0.0012	13	6	1.83 × 10^−5^	1.46 × 10^−4^	*SMC3*,*RAD21*,*PLK1*,*SMC1A*,*WAPL*,*PDS5A*	Cell Cycle	Cell Cycle, Mitotic	Mitotic Telophase/Cytokinesis
Removal of the Flap Intermediate	0.0013	14	6	2.75 × 10^−5^	1.93 × 10^−4^	*PCNA*,*RPA1*,*RPA2*,*RPA3*,*POLD3*,*POLD2*	Cell Cycle	Cell Cycle, Mitotic	Removal of the Flap Intermediate
G1/S Transition	0.0118	131	17	3.50 × 10^−5^	2.45 × 10^−4^	*MYC*,*AKT1*,*PSME1*,*PSMC6*,*PSMC3*,*MCM7*,*MCM6*,*MCM2*,*HDAC1*,*RBBP4*,*SKP2*,*PCNA*,*RPA1*,*RPA2*,*RPA3*,*CCND1*,*PSMD11*	Cell Cycle	Cell Cycle, Mitotic	G1/S Transition
Processive Synthesis on the Lagging Strand	0.0014	15	6	4.03 × 10^−5^	2.82 × 10^−4^	*PCNA*,*RPA1*,*RPA2*,*RPA3*,*POLD3*,*POLD2*	Cell Cycle	Cell Cycle, Mitotic	Processive Synthesis on the Lagging Strand
Depolymerization of the Nuclear Lamina	0.0014	15	6	4.03 × 10^−5^	2.82 × 10^−4^	*PRKCA*,*EMD*,*LEMD2*,*TMPO*,*LMNA*,*LMNB1*	Cell Cycle	Cell Cycle, Mitotic	Depolymerization of the Nuclear Lamina
Mitotic G1 phase and G1/S transition	0.0134	149	18	5.17 × 10^−5^	3.29 × 10^−4^	*MYC*,*AKT1*,*PSME1*,*PSMC6*,*PSMC3*,*MCM7*,*MCM6*,*MCM2*,*HDAC1*,*RBBP4*,*SKP2*,*TOP2A*,*PCNA*,*RPA1*,*RPA2*,*RPA3*,*CCND1*,*PSMD11*	Cell Cycle	Cell Cycle, Mitotic	Mitotic G1 Phase and G1/S Transition
Cohesin Loading Onto Chromatin	0.0009	10	5	6.34 × 10^−5^	3.81 × 10^−4^	*SMC3*,*RAD21*,*SMC1A*,*WAPL*,*PDS5A*	Cell Cycle	Cell Cycle, Mitotic	Cohesin Loading onto Chromatin
Establishment of Sister Chromatid Cohesion	0.001	11	5	9.88 × 10^−5^	5.93 × 10^−4^	*SMC3*,*RAD21*,*SMC1A*,*WAPL*,*PDS5A*	Cell Cycle	Cell Cycle, Mitotic	Establishment of Sister Chromatid Cohesion

**Table 4:** The 28 top-hit longevity pathways are shown. The general, specific, and more pathways were traced for the Reactome pathways. All of the pathways in Table 4 fall under the gene expression (transcription) or signal transduction pathways.

Selected Pathways for Longevity Pathway Enrichment									
Reactome Pathway	Ratio of Protein in Pathway	Number of Proteins in Pathway	Protein from Gene Set	*p*-Value	FDR	Hit Genes	General	Specific	More Specific
MTOR signaling	0.0037	41	19	1.11 × 10^−16^	5.34 × 10^−14^	*AKT1*,*RRAGA*,*RRAGC*,*RRAGB*,*RRAGD*,*RPTOR*,*LAMTOR2*,*LAMTOR3*,*RPS6*,*TSC2*,*TSC1*,*EIF4EBP1*,*RHEB*,*MLST8*,*AKT1S1*,*EIF4E*,*EIF4B*,*MTOR*,*RPS6KB1*	Signal transduction	MTOR signaling	
mTORC1-mediated signaling	0.0022	24	16	1.11 × 10^−16^	5.34 × 10^−14^	*RRAGA*,*RRAGC*,*RRAGB*,*RRAGD*,*RPTOR*,*LAMTOR2*,*LAMTOR3*,*RPS6*,*EIF4EBP1*,*RHEB*,*MLST8*,*AKT1S1*,*EIF4E*,*EIF4B*,*MTOR*,*RPS6KB1*	Signal transduction	MTOR signaling	mTORC1-mediated signaling
Intracellular signaling by second messengers	0.0294	326	34	1.29 × 10^−14^	4.12 × 10^−12^	*AKT1*,*PRKCA*,*RRAGA*,*RRAGC*,*RRAGB*,*RRAGD*,*TP53*,*MAPKAP1*,*TGFA*,*INSR*,*CDKN1A*,*RPTOR*,*PPARG*,*EGFR*,*RICTOR*,*CAMK4*,*LAMTOR2*,*LAMTOR3*,*IRS2*,*TSC2*,*FOXO4*,*FOXO3*,*FOXO1*,*KL*,*ESR1*,*NBEA*,*RHEB*,*FGFR1*,*PRR5*,*MLST8*,*PIK3CA*,*AKT1S1*,*INS*,*MTOR*	Signal transduction	Intracellular signaling by second messengers	
PIP3 activates AKT signaling	0.0258	286	31	7.83 × 10^−14^	1.88 × 10^−11^	*AKT1*,*RRAGA*,*RRAGC*,*RRAGB*,*RRAGD*,*TP53*,*MAPKAP1*,*TGFA*,*INSR*,*CDKN1A*,*RPTOR*,*PPARG*,*EGFR*,*RICTOR*,*LAMTOR2*,*LAMTOR3*,*IRS2*,*TSC2*,*FOXO4*,*FOXO3*,*FOXO1*,*KL*,*ESR1*,*RHEB*,*FGFR1*,*PRR5*,*MLST8*,*PIK3CA*,*AKT1S1*,*INS*,*MTOR*	Signal transduction	Intracellular signaling by second messengers	PIP3 activates AKT signaling
Generic transcription pathway	0.1113	1235	66	2.29 × 10^−13^	4.40 × 10^−11^	*SERPINE1*,*AKT1*,*RUNX3*,*RRAGA*,*RRAGC*,*RRAGB*,*RRAGD*,*TP53*,*MAPKAP1*,*TGFA*,*GATA4*,*APOE*,*ATRIP*,*PPARGC1A*,*ATM*,*CDKN1A*,*MSTN*,*RPTOR*,*SREBF1*,*SIRT1*,*SIRT3*,*RAD51D*,*RARB*,*PPARG*,*SGK1*,*EGFR*,*WRN*,*RICTOR*,*WWOX*,*CDKN2B*,*GSR*,*MLH1*,*VEGFA*,*CAMK4*,*FAS*,*LAMTOR2*,*LAMTOR3*,*NR3C1*,*YY1*,*KCTD1*,*TSC2*,*TSC1*,*TBL1XR1*,*CSF1R*,*FOXO4*,*FOXO3*,*FOXO1*,*PLXNA4*,*TGFB1*,*ESRRG*,*ESR1*,*NFKB1*,*IL6*,*CDK6*,*RHEB*,*PRR5*,*EXO1*,*MLST8*,*YWHAG*,*H2AFX*,*IFNG*,*INS*,*TXNRD1*,*SOD2*,*MTOR*,*ERCC2*	Gene expression (transcription)	RNA polymerase II transcription	Generic transcription pathway
Energy-dependent regulation of mTOR by LKB1-AMPK	0.0026	29	12	1.95 × 10^−12^	3.12 × 10^−10^	*RRAGA*,*RRAGC*,*RRAGB*,*RRAGD*,*RPTOR*,*LAMTOR2*,*LAMTOR3*,*TSC2*,*TSC1*,*RHEB*,*MLST8*,*MTOR*	Signal transduction	MTOR signaling	Energy-dependent regulation of mTOR by LKB1-AMPK
RNA polymerase II transcription	0.1229	1364	67	6.72 × 10^−12^	9.20 × 10^−10^	*SERPINE1*,*AKT1*,*RUNX3*,*RRAGA*,*RRAGC*,*RRAGB*,*RRAGD*,*TP53*,*MAPKAP1*,*TGFA*,*GATA4*,*APOE*,*ATRIP*,*PPARGC1A*,*ATM*,*CDKN1A*,*MSTN*,*RPTOR*,*SREBF1*,*SIRT1*,*SIRT3*,*RAD51D*,*RARB*,*PPARG*,*SGK1*,*EGFR*,*WRN*,*RICTOR*,*WWOX*,*CDKN2B*,*GSR*,*MLH1*,*VEGFA*,*CAMK4*,*FAS*,*LAMTOR2*,*LAMTOR3*,*NR3C1*,*YY1*,*KCTD1*,*TSC2*,*TSC1*,*TBL1XR1*,*CSF1R*,*FOXO4*,*FOXO3*,*FOXO1*,*PLXNA4*,*TGFB1*,*ESRRG*,*ESR1*,*NFKB1*,*IL6*,*CDK6*,*RHEB*,*PRR5*,*EXO1*,*MLST8*,*YWHAG*,*H2AFX*,*IFNG*,*POLDIP3*,*INS*,*TXNRD1*,*SOD2*,*MTOR*,*ERCC2*	Gene expression (transcription)	RNA polymerase II transcription	
TP53 regulates metabolic genes	0.008	89	17	9.43 × 10^−12^	1.13 × 10^−9^	*AKT1*,*RRAGA*,*RRAGC*,*RRAGB*,*RRAGD*,*TP53*,*RPTOR*,*GSR*,*LAMTOR2*,*LAMTOR3*,*TSC2*,*TSC1*,*RHEB*,*MLST8*,*YWHAG*,*TXNRD1*,*MTOR*	Gene expression (transcription)	RNA polymerase II transcription	Generic transcription pathway
FOXO-mediated transcription	0.006	67	15	1.84 × 10^−11^	1.95 × 10^−9^	*AKT1*,*PPARGC1A*,*CDKN1A*,*MSTN*,*SREBF1*,*SIRT1*,*SIRT3*,*NR3C1*,*FOXO4*,*FOXO3*,*FOXO1*,*PLXNA4*,*YWHAG*,*INS*,*SOD2*	Gene expression (transcription)	RNA polymerase II transcription	Generic transcription pathway
Transcriptional regulation by TP53	0.0332	368	30	2.09 × 10^−10^	1.82 × 10^−8^	*AKT1*,*RRAGA*,*RRAGC*,*RRAGB*,*RRAGD*,*TP53*,*MAPKAP1*,*ATRIP*,*ATM*,*CDKN1A*,*RPTOR*,*RAD51D*,*SGK1*,*WRN*,*RICTOR*,*GSR*,*MLH1*,*FAS*,*LAMTOR2*,*LAMTOR3*,*TSC2*,*TSC1*,*RHEB*,*PRR5*,*EXO1*,*MLST8*,*YWHAG*,*TXNRD1*,*MTOR*,*ERCC2*	Gene expression (transcription)	RNA polymerase II transcription	Generic transcription pathway
FOXO-mediated transcription of oxidative stress and metabolic and neuronal genes	0.0028	31	10	1.49 × 10^−9^	1.19 × 10^−7^	*PPARGC1A*,*SREBF1*,*SIRT3*,*NR3C1*,*FOXO4*,*FOXO3*,*FOXO1*,*PLXNA4*,*INS*,*SOD2*	Gene expression (transcription)	RNA polymerase II transcription	Generic transcription pathway
Regulation of PTEN gene transcription	0.0055	61	12	8.21 × 10^−9^	5.58 × 10^−7^	*RRAGA*,*RRAGC*,*RRAGB*,*RRAGD*,*TP53*,*RPTOR*,*PPARG*,*LAMTOR2*,*LAMTOR3*,*RHEB*,*MLST8*,*MTOR*	Signal transduction	Intracellular signaling by second messengers	PIP3 activates AKT signaling
Transcriptional regulation by the AP-2 (TFAP2) family of transcription factors	0.0032	36	9	8.53 × 10^−8^	4.78 × 10^−6^	*TGFA*,*APOE*,*CDKN1A*,*EGFR*,*WWOX*,*VEGFA*,*YY1*,*KCTD1*,*ESR1*	Gene expression (transcription)	RNA polymerase II transcription	Generic transcription pathway
Regulation of TP53 degradation	0.0032	36	9	8.53 × 10^−8^	4.78 × 10^−6^	*AKT1*,*TP53*,*MAPKAP1*,*ATM*,*SGK1*,*RICTOR*,*PRR5*,*MLST8*,*MTOR*	Gene expression (transcription)	RNA polymerase II transcription	Generic transcription pathway
Regulation of TP53 expression and degradation	0.0033	37	9	1.07 × 10^−7^	5.69 × 10^−6^	*AKT1*,*TP53*,*MAPKAP1*,*ATM*,*SGK1*,*RICTOR*,*PRR5*,*MLST8*,*MTOR*	Gene expression (transcription)	RNA polymerase II transcription	Generic transcription pathway
VEGFA-VEGFR2 pathway	0.0086	95	12	9.06 × 10^−7^	3.35 × 10^−5^	*AKT1*,*PRKCA*,*MAPKAP1*,*HRAS*,*RHOA*,*RICTOR*,*VEGFA*,*PRR5*,*ITGB3*,*MLST8*,*PIK3CA*,*MTOR*	Signal transduction	Signaling by receptor tyrosine kinases	Signaling by VEGF
Signaling by VEGF	0.0094	104	12	2.28 × 10^−6^	7.99 × 10^−5^	*AKT1*,*PRKCA*,*MAPKAP1*,*HRAS*,*RHOA*,*RICTOR*,*VEGFA*,*PRR5*,*ITGB3*,*MLST8*,*PIK3CA*,*MTOR*	Signal transduction	Signaling by receptor tyrosine kinases	Signaling by VEGF
Regulation of FOXO transcriptional activity by acetylation	0.0009	10	5	2.52 × 10^−6^	8.33 × 10^−5^	*SIRT1*,*SIRT3*,*FOXO4*,*FOXO3*,*FOXO1*	Gene expression (transcription)	RNA polymerase II transcription	Generic transcription pathway
Regulation of localization of FOXO transcription factors	0.0011	12	5	6.07 × 10^−6^	1.76 × 10^−4^	*AKT1*,*FOXO4*,*FOXO3*,*FOXO1*,*YWHAG*	Gene expression (transcription)	RNA polymerase II transcription	Generic transcription pathway
Signaling by receptor tyrosine kinases	0.0458	508	27	7.81 × 10^−6^	2.19 × 10^−4^	*AKT1*,*PRKCA*,*MAPKAP1*,*TGFA*,*HIF1A*,*APOE*,*LYN*,*INSR*,*IGF2*,*HRAS*,*RHOA*,*SGK1*,*EGFR*,*RICTOR*,*WWOX*,*VEGFA*,*IRS2*,*IGF1R*,*KL*,*ESR1*,*FGFR1*,*PRR5*,*ITGB3*,*MLST8*,*PIK3CA*,*INS*,*MTOR*	Signal transduction	Signaling by receptor tyrosine kinases	
TFAP2 (AP-2) family regulates transcription of growth factors and their receptors	0.0012	13	5	8.91 × 10^−6^	2.22 × 10^−4^	*TGFA*,*EGFR*,*VEGFA*,*YY1*,*ESR1*	Gene expression (transcription)	RNA polymerase II transcription	Generic transcription pathway
RUNX3 regulates CDKN1A transcription	0.0006	7	4	1.58 × 10^−5^	3.63 × 10^−4^	*RUNX3*,*TP53*,*CDKN1A*,*TGFB1*	Gene expression (transcription)	RNA polymerase II transcription	Generic transcription pathway
VEGFR2 mediated vascular permeability	0.0024	27	6	2.45 × 10^−5^	5.39 × 10^−4^	*AKT1*,*MAPKAP1*,*RICTOR*,*PRR5*,*MLST8*,*MTOR*	Signal transduction	Signaling by receptor tyrosine kinases	Signaling by VEGF
PTEN regulation	0.0142	158	13	3.09 × 10^−5^	6.69 × 10^−4^	*AKT1*,*RRAGA*,*RRAGC*,*RRAGB*,*RRAGD*,*TP53*,*RPTOR*,*PPARG*,*LAMTOR2*,*LAMTOR3*,*RHEB*,*MLST8*,*MTOR*	Signal transduction	Intracellular signaling by second messengers	PIP3 activates AKT signaling
FOXO-mediated transcription of cell cycle genes	0.0015	17	5	3.19 × 10^−5^	6.69 × 10^−4^	*CDKN1A*,*MSTN*,*FOXO4*,*FOXO3*,*FOXO1*	Gene expression (transcription)	RNA polymerase II transcription	Generic transcription pathway
Signaling by nuclear receptors	0.0245	272	17	6.09 × 10^−5^	1.22 × 10^−3^	*AKT1*,*CETP*,*TGFA*,*APOE*,*HRAS*,*SREBF1*,*RARB*,*DLD*,*EGFR*,*IGF1R*,*YY1*,*TBL1XR1*,*FOXO3*,*ESR1*,*H2AFX*,*PIK3CA*,*APOC1*	Signal transduction	Signaling by nuclear receptors	
AKT phosphorylates targets in the nucleus	0.0009	10	4	6.26 × 10^−5^	1.22 × 10^−3^	*AKT1*,*FOXO4*,*FOXO3*,*FOXO1*	Signal transduction	Intracellular signaling by second messengers	PIP3 activates AKT signaling
PI5P, PP2A, and IER3 regulate PI3K/AKT signaling	0.0096	106	10	8.53 × 10^−5^	1.62 × 10^−3^	*AKT1*,*TGFA*,*INSR*,*EGFR*,*IRS2*,*KL*,*ESR1*,*FGFR1*,*PIK3CA*,*INS*	Signal transduction	Intracellular signaling by second messengers	PIP3 activates AKT signaling

## Data Availability

All of the data have been included in the manuscript.

## Supplementary Materials

The following supporting information can be downloaded at https://www.mdpi.com/article/10.3390/ijms251910493/s1.
